# LACKING FAMILY TIES DURING A GLOBAL PANDEMIC: OLDER ADULTS CONCEPTUALIZATIONS OF SOCIAL SUPPORT

**DOI:** 10.1093/geroni/igac059.2977

**Published:** 2022-12-20

**Authors:** Bryce Van Vleet, Emily Kinkade, Heather Fuller, Andrea Huseth-Zosel

**Affiliations:** North Dakota State University, Fargo, North Dakota, United States; North Dakota State University, Fargo, North Dakota, United States; North Dakota State University, Fargo, North Dakota, United States; North Dakota State University, Fargo, North Dakota, United States

## Abstract

Social support from family provided benefits for coping during the COVID-19 pandemic; however, not all older adults had access to family. The present study investigates how older adults without access to traditional family ties conceptualized their social relationships throughout the first two years of the pandemic. A subsample of eight older adults without direct access to traditional family ties were identified from a larger 5-wave interview study conducted between April 2020 and June 2022. Transcripts were holistically coded and three overarching themes emerged: constraints of place, redefining family, and feelings of isolation and closeness. The first theme addressed having family members living far away and uncertainty of when they would get to see them again. However, these distance barriers could be overcome through technology. The second theme illuminated that during the pandemic, those without access to traditional family ties redefined their social relationships by developing fictive kin from neighbors, colleagues, and friends. The third theme highlighted that some older adults felt they were lacking strong social networks and were concerned they had nobody to contact if they needed help, while others felt that despite limitations, their social relationships grew closer due to connection through alternative forms of communication (e.g. texting). Results from this study clarify how traditional family ties were challenged and strengthened during physical distancing for some older adults. These findings extend the literature on how fictive kin forms in older adulthood during temporary crises and suggests potential avenues for social connection for older adults lacking traditional family support.

